# Role and Regulation of Pif1 Family Helicases at the Replication Fork

**DOI:** 10.3390/ijms23073736

**Published:** 2022-03-29

**Authors:** Emory G. Malone, Matthew D. Thompson, Alicia K. Byrd

**Affiliations:** 1 Department of Biochemistry and Molecular Biology, University of Arkansas for Medical Sciences, Little Rock, AR 72205, USA; emalone2@uams.edu (E.G.M.); mdthompson@uams.edu (M.D.T.); 2 Winthrop P. Rockefeller Cancer Institute, University of Arkansas for Medical Sciences, Little Rock, AR 72205, USA

**Keywords:** Pif1 helicase, DNA helicase, replication fork, replication fork barriers, G-quadruplexes, telomerase

## Abstract

Pif1 helicases are a multifunctional family of DNA helicases that are important for many aspects of genomic stability in the nucleus and mitochondria. Pif1 helicases are conserved from bacteria to humans. Pif1 helicases play multiple roles at the replication fork, including promoting replication through many barriers such as G-quadruplex DNA, the rDNA replication fork barrier, tRNA genes, and R-loops. Pif1 helicases also regulate telomerase and promote replication termination, Okazaki fragment maturation, and break-induced replication. This review highlights many of the roles and regulations of Pif1 at the replication fork that promote cellular health and viability.

## 1. Introduction to Pif1 Helicases

DNA helicases utilize the energy of NTP hydrolysis to translocate on and unwind DNA for processes such as replication and repair. The Pif1 helicase family is part of helicase superfamily 1B [1], which translocates on single-stranded DNA (ssDNA) and unwinds duplex DNA in the 5′-to-3′ direction [2]. Pif1 is conserved in all eukaryotes and some bacteria [1]. *Saccharomyces cerevisiae* Pif1 (ScPif1) is the prototypical member of the family. ScPif1 was discovered when its deletion resulted in the loss of mitochondrial DNA and respiratory-deficient (petite) cells, coining its name: petite integration frequency (Pif1) [3,4].

*S. cerevisiae* encodes two different Pif1 helicases, ScPif1 and Rrm3, which both localize to the nucleus and mitochondria [5,6,7] Both isoforms of ScPif1 are encoded from a single open reading frame, such that translation beginning at the first start codon (M1) results in nuclear ScPif1 and translation beginning at the second start codon (M40) results in mitochondrial ScPif1 [8]. In fact, many assays for either nuclear or mitochondrial ScPif1 will use *pif1-m1* or *pif1-m2* alleles with point mutations in the first or second start codon that result in the expression of nuclear ScPif1 or mitochondrial ScPif1, respectively [9]. The use of *pif1-m2* cells to ascertain the function of nuclear ScPif1 without the mitochondrial defects associated with ScPif1 loss is common, although nuclear effects are less drastic in *pif1-m2* cells than in *pif1Δ* cells, suggesting that a small amount of mitochondrial ScPif1 leaks into the nucleus [10]. In addition to *S. cerevisiae*, some other fungi also encode two Pif1 helicases, but encoding two Pif1 proteins is not an innate trait of yeast genomes since *Schizosacchromyces pombe* encodes for only one Pif1 helicase, Pfh1, which stabilizes both mitochondrial and nuclear DNA [11]. Although Pif1 is nonessential in *S. cerevisiae*, Pfh1 is essential in *S. pombe* [12]. Most multicellular organisms also encode only one Pif1 family helicase. The human PIF1 (hPIF1) has two splice variants: one that localizes to the nucleus and another that localizes to the mitochondria [13]. Regardless of the number, Pif1 family helicases seem to localize to both the nucleus and the mitochondria [1].

The Pif1 signature motif (DKLeXvARaiRKqXkPFGGIQli) between helicase motifs II and III [14] differentiates Pif1 and other superfamily 1B helicases [15,16]. The first 10 amino acids of the signature motif form an α-helix and the following 11 amino acids form an extended loop [16]. While the signature motif is conserved through bacteria, yeast, and humans, it is not always conserved in Pif1 in plants [16]. The signature motif stabilizes the structure [17], is essential for the DNA binding and ATPase activities of ScPif1 [16,17], and is essential for DNA unwinding and protein displacement by Pfh1 [18]. Not surprisingly, the signature motif is required for many in vivo ScPif1 activities, including its mitochondrial functions, the removal of telomerase from telomeres and double-stranded DNA breaks (DSBs), and Okazaki fragment processing and maturation [16]. A point mutation in the signature motif, L319P, in hPIF1 is associated with an increased risk of breast cancer [19] and likely causes a kink in an α-helix that destabilizes the protein [17]. Mohammad et al. engineered three point mutations in the corresponding amino acids of nuclear Pfh1 (L430P, L430V, L430A) and found that the mutations L430P and L430V exhibited impaired ATP hydrolysis [18]. Perhaps it is no wonder that a point mutation in the hPif1 signature motif can increase the risk of breast cancer [19], since Pif1 and the Pif1 signature motif play a major role in genetic stability.

## 2. Pif1 Structure

ScPif1, Rrm3, Pfh1, and hPIF1 have three domains: the N-terminal domain (NTD), the central helicase domain, and the C-terminal domain (CTD) [20,21,22,23]. However, only the helicase domain is conserved [20], and the ScPif1 and Rrm3 helicase domains are interchangeable [23]. They are responsible for binding and hydrolyzing ATP and binding and translocating on ssDNA to unwind duplex DNA, unfold G-quadruplex DNA, and displace proteins bound to DNA [17,24,25,26]. The NTDs of ScPif1, Rrm3, and Pfh1 all contain mitochondrial localization sequences [27], whereas hPIF1 contains a mitochondrial localization sequence in the CTD [13]. The NTD and CTD accessory domains have been proposed to function as sites of protein–protein interactions, [28], posttranslational modification [8], and regulation [29], and as providers of secondary activities such as strand annealing [30]. Recent evidence suggests these domains may also directly modulate helicase activity [23].

**Figure 1:**
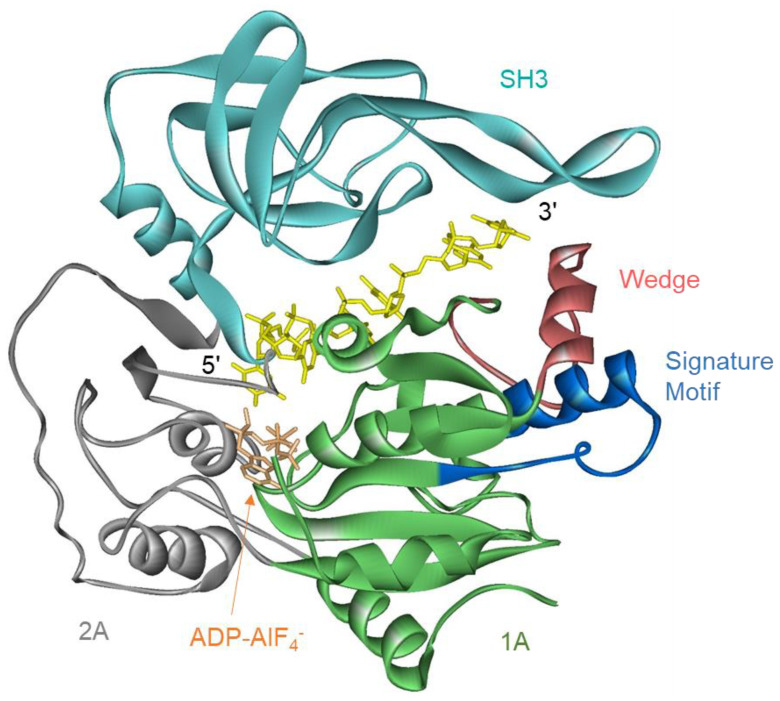
Structure of BaPif1 (PDB ID: 5FHE [17]). The Pif1 signature motif (blue) provides structural support for the strand separation wedge (pink). The signature motif and wedge are insertions within domain 1A (green). Domains 1A and 2A (gray) are RecA-like domains that are conserved in all helicases. The SH3 domain (cyan) is an insertion within domain 2A. The bound DNA is yellow and ADP-AlF_4_^−^ is orange.

Structures of the helicase domain of ScPif1 and hPIF1 and of two bacterial Pif1s which consist of only a helicase domain, *Bacteroides* spp. (BsPif1) and *Bacteroides* sp. 2-1-16 (BaPif1), are available [17,25,31,32]. All share a similar structure comprising two RecA-like domains containing the conserved helicase motifs, an SH3 domain, and a wedge domain for separating the incoming duplex. The wedge is stabilized by the Pif1 signature motif (Figure 1) [17].

The mutation of the ATPase active site abolishes helicase activity but not its strand-annealing ability [18,30,33]. The deletion of the ScPif1 NTD results in greater affinity for ssDNA than full-length ScPif1, suggesting that the NTD inhibits binding to single-stranded nucleic acids [29]. Full-length ScPif1 preferentially unwinds RNA-DNA hybrid duplexes with the RNA in the displaced strand [34] due to increased processivity on the RNA-DNA duplex [35], but this enhanced activity on RNA-DNA duplexes is lost when the NTD is removed [29]. The NTD of ScPif1 is required in order for the mitochondrial single-stranded binding protein, Rim1, to enhance the unwinding activity of ScPif1 [36], even though direct interactions between the helicase domain of ScPif1 and Rim1 have been reported [37]. The strand annealing activity of hPIF1 resides in the NTD [30]. The NTD of Rrm3 is required for interaction with Orc5 [28] and PCNA [38] and is necessary in order for Rrm3 function in vivo [27]. A chimeric protein containing the NTD of Rrm3 and the helicase domain of ScPif1 or BaPif1 can substitute for Rrm3 in vivo [23].

A structure of a non-canonical PCNA-interacting protein (PIP) box in the CTD of ScPif1 interacting with PCNA has been solved [39]. The mutation of this sequence reduces strand displacement synthesis by Polδ-PCNA-ScPif1 and results in defects in break-induced replication [39]. However, ScPif1 also has two canonical PIP boxes in the helicase domain [40]. The PIP box, which is present on the C-terminus of the helicase domain of ScPif1, also interacts with PCNA and is important, but not required, for replication through lagging-strand G-quadruplex structures [40]. The non-canonical PIP box in the CTD has no effect on replication through G-quadruplex structures. Whether the second PIP box in the helicase domain interacts with PCNA and allows Pif1 to carry out some of its functions is unknown. However, ScPif1 appears to interact with PCNA in at least two different sites. Pfh1 also interacts with PCNA through a PIP box in its NTD [38].

The C-terminal domain of *Thermotoga elfii* Pif1 increases the affinity for ssDNA by providing a secondary ssDNA binding site in addition to the DNA binding site in the helicase domain [23]. This is critical for coupling ATP hydrolysis to DNA unwinding, as the loss of the CTD domain ssDNA binding site leads to increased ATPase rates but reduced DNA unwinding rates [23]. These observations suggest that, in addition to functions in protein–protein interactions and oligomerization, the N- and C-terminal accessory domains can modulate the enzymatic activity of Pif1 helicases.

**Figure 2:**
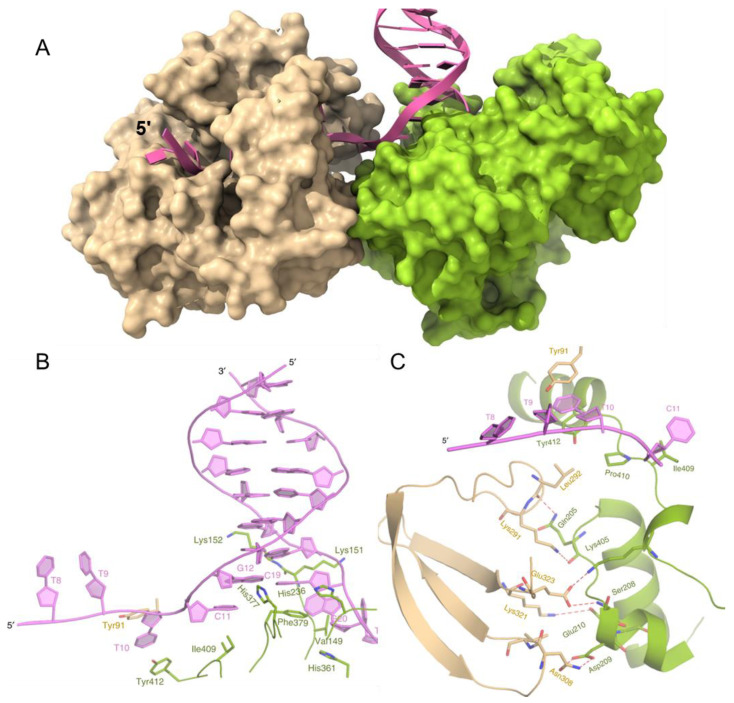
Structure of BaPif1 bound to a forked DNA. (**A**) Two molecules of BaPif1 (tan and green) are bound to the DNA fork (magenta). (**B**) The BaPif1 on the 3′-arm of the fork (green) is bound at the junction. (**C**) The two BaPif1 molecules interact. PDB ID 6L3G. Images reproduced from reference [26] Creative Commons CC BY.

Recently, a structure of BaPif1 bound to a forked duplex was reported [26]. Two BaPif1 molecules are bound, one to each arm of the fork (Figure 2A). Surprisingly, the BaPif1 on the 3′-arm of the fork is positioned at the ssDNA–dsDNA junction, where it stabilizes a separated base pair. As Pif1 helicases unwind DNA 5′-to-3′, this enzyme is not positioned to unwind the duplex but instead appears to block access to the fork by the BaPif1 bound to the 5′-arm of the fork that is positioned back from the junction (Figure 2A,B). However, both BaPif1 molecules are active helicases and can unwind a substrate with two duplexes: one to report on the activity of the 5′-Pif1 and one to simultaneously report on the activity of the 3′-Pif1 [26]. The two BaPif1 molecules interact (Figure 2C) and appear to regulate each other as both Pif1 molecules unwind duplex DNA faster and are more processive when an electrostatic interaction at the interface between the molecules is interrupted by the mutation of Glu323. This limitation of activity may serve to constrain Pif1 to local regions such as G-quadruplexes or protein-bound DNA, where it may repetitively resolve structures present without unwinding large portions of the genome. Many helicases, including ScPif1, have been shown through single-molecule FRET (smFRET) to repetitively and transiently unwind duplexes, unfold G4 structures, and remove bound proteins [41,42,43,44,45]. ScPif1 has also been observed to form DNA loops during translocation on ssDNA using magnetic tweezers [46]. An interaction of two Pif1 molecules at the fork where each constrains the activity of the other while repetitively looping DNA through to remove an obstacle at the fork is an attractive model to describe how some Pif1 helicases may limit their activity to local regions of the DNA. However, whether this structure is common to all Pif1 family helicases or is specific to BaPif1 is unknown. It is difficult to imagine how this mechanism could be beneficial for Rrm3 and Pfh1 because they travel with the replisome [47,48].

## 3. Pif1 Resolves Replication Barriers

Pif1 helicases have multifunctional roles in the cell and at the replication fork. Pif1 helicases localize to and enhance the progression of the replication fork through many types of structures that may disrupt replication and lead to DNA damage in the absence of Pif1, including G-quadruplex structures, R-loops, and protein-bound DNA. Some Pif1 family helicases such as Rrm3 and Pfh1 are associated with the replisome, where they aid in progression of the fork [47,48].

### 3.1. G-Quadruplex Structures

G-quadruplexes (G4) are secondary structures in DNA that form from intra- or intermolecular guanosine hydrogen bonding, forming highly stable planar tetrads stabilized by Hoogsteen hydrogen bonds [49]. G4 DNA structures regulate transcription, translation, telomere maintenance, and immunoglobulin heavy chain isotype switching [50]. G4 DNA is enriched in telomeres [51], meiotic and mitotic DSB hotspots [52], mitochondrial DNA deletion breakpoints [53], gene promoters [54], ribosomal DNA (rDNA) [55], and untranslated regions (UTRs) [56]. The difficulty experienced in unwinding and replicating these regions makes them a source of genomic instability [57,58]. However, multiple Pif1 family helicases can reduce genomic instability at G4 DNA motifs.

Pfh1 and ScPif1 preferentially bind to G4 sequences in the genome and promote genomic stability at these sites [59,60,61,62]. ScPif1, Pfh1, and hPIF1 all unfold G4 DNA structures in vitro [42,43,63,64,65], and ScPif1 promotes DNA synthesis through G4 DNA structures by Polδ, POLγ, and Mip1 (the *S. cerevisiae* POLγ homolog) [40,66,67]. In cells lacking either ScPif1 or Pfh1, there is increased replication pausing and the breakage of forks at G4 motifs [59,61]. In the absence of ScPif1, G4-containing microsatellites are unstable, and mutations and gross chromosomal rearrangements accumulate around G4s [58,60,61,62]. DNA damage also accumulates around G4 motifs in Pfh1-deficient *S. pombe* as the phosphorylation of histone H2A, producing γ-H2A, occurs at G4 motifs but not at regions with only a high GC content that could not form G4 secondary structures [59]. The ability of cells to conduct DNA replication at sites with G4 DNA is dependent on the stability of the quadruplex [40,67,68], where shorter G4 structures tend to be more thermally stable [62,67,68,69]. G4 DNA can fold into parallel and antiparallel structures, where parallel structures are the preferred form in *S. cerevisiae* and tend to also have greater thermal stability [70]. However, because these structures are more stable, it is more difficult for Pif1 to unwind them [71].

**Figure 3:**
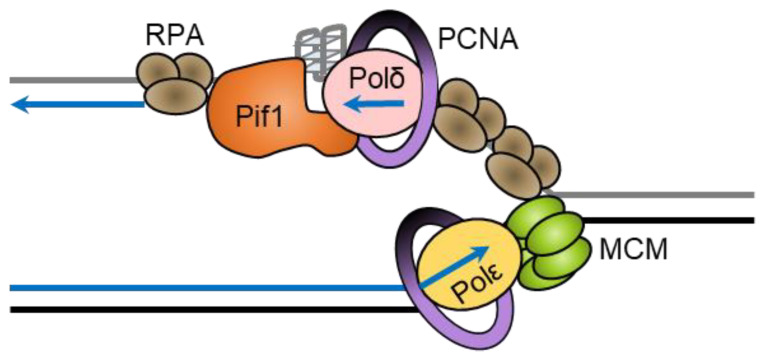
ScPif1 stimulates replication through lagging-strand G-quadruplexes. The leading strand is synthesized by Polε (yellow) in complex with PCNA (purple) at the same time as duplex unwinding by CMG (green). RPA (brown) may not be able to prevent G4 structures from forming on the lagging strand, so Pif1 (orange) may unfold G4 structures so that Polδ (pink) can synthesize the lagging strand in complex with PCNA. Since Polδ translocates 3′-to-5′ on the template and Pif1 translocates 5′-to-3′, Pif1 would approach the G4 structure from the opposite side to that of Polδ.

Interestingly, Pif1 cooperates with the single-stranded binding protein replication protein A (RPA) to resolve G4 structures on both the leading and lagging strand during DNA replication [72]. Human RPA unfolds less stable G4 structures on its own, but structures with loops less than three nucleotides long or with four or more tetrads are resistant to unfolding by RPA [73]. ScPif1 is particularly important for replication through lagging-strand G4 DNA sequences [40]. This stimulation of replication through G4 DNA sequences requires the interaction of ScPif1 with PCNA through a canonical PIP box in the helicase domain of ScPif1 [40]. The effect on the replication rate is seen only when the G4 DNA sequence is on the lagging strand, suggesting that RPA may not prevent the folding of G4 DNA structures in the ssDNA on the lagging strand. ScPif1 could then be necessary in order to unfold the G4 DNA structure to allow synthesis by Polδ (Figure 3). Since Pif1 moves in the 5′-to-3′ direction, a head-on collision with DNA polymerase δ may occur as the polymerase translocates along the template in the 3′-to-5′ direction [67]. After a head-on collision between Pif1 helicase and a polymerase, the polymerase’s exonuclease activity may become active. However, single-strand binding proteins may act as a bumper to prevent primer degradation from the polymerase’s exonuclease activity [67].

hPIF1 interacts with BRCA1 to facilitate the resection of G4 sequences during DSB repair by homologous recombination [74]. Rrm3 is required for the repair of DSBs that form due to replication fork breakage [75]. Although it is possible that, like hPIF1, Rrm3 promotes resection across G4 sequences, this seems unlikely because Rrm3 has not been reported to have G4 DNA unfolding activity. *Drosophila* PIF1 is critical for genome maintenance and the survival of embryos exposed to the replication stalling agent hydroxyurea [76]. As G4 structures contribute to replication fork stalling, this suggests that Pif1 family helicases may also respond to replication fork stalling at G4 structures in addition to promoting replication through these structures.

### 3.2. R-Loops

**Figure 4:**
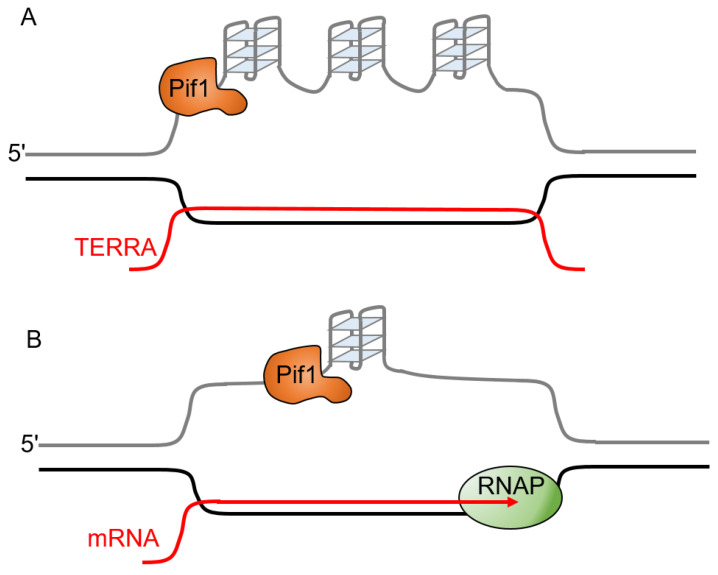
The role of Pif1 at R-loops may be to resolve G4 structures that form on the displaced strand. (**A**) Multiple G4 structures can form on the displaced strand of a telomeric R-loop. (**B**) G4 formation on the displaced strand of a co-transcriptional R-loop is likely to be more limited. In either case, Pif1 (orange triangle) is proposed to resolve the G4 structures on the displaced strand instead of resolving the R-loop itself. Green color shows RNA polymerase.

The formation of RNA:DNA hybrids within duplex DNA during transcription results in the formation of structures called R-loops that can potentially cause genetic instability [77,78]. Their formation is normally transient, as they can be resolved by RNase H that degrades the RNA in RNA:DNA hybrids. R-loops can form between newly transcribed RNA and the template DNA strand. In addition to formation at actively transcribed genes, R-loops also form at telomeres when the G-rich telomere repeat-containing RNA (TERRA) base pairs with telomeric DNA [79]. These telomeric R-loops increase chromosome fragility [80]. In the case of telomeric R-loops, both the R-loop and the potential for G-quadruplex formation on the displaced G-rich strand (Figure 4A) have the potential to stall replication forks [79]. Since G-quadruplexes and R-loops can both promote the formation of each other, they may cooperate to increase the rate of replication fork stalling [81,82,83,84]. G4 formation in the displaced strand of the R-loop (Figure 4B) results in an increase in markers of DNA damage and can cause replication stress [81]. Conflicts between the transcription and replication machinery can allow G4 structures to form on the non-template strand, leading to the stalling of the leading strand polymerase and a gap in the lagging strand [85]. ScPif1 reduces the formation of lagging-strand gaps at co-directional R-loops in the presence of RNase H1 [85]. RNase H1 is sufficient to resolve R-loops and ScPif1 does not enhance fork progression through co-directional R-loops, likely because the limited processivity of ScPif1 prevents it from resolving the long RNA:DNA hybrids that form during transcription [85]. Although the processivity of ScPif1 is greater in RNA:DNA hybrid duplexes than in DNA:DNA duplexes, ScPif1 is only able to unwind 15–20 base pairs of a RNA:DNA duplex in a single binding event [35]. This suggests that the role of ScPif1 at R-loops may be to resolve the G4 DNA structures that form on the displaced (non-template) strand as opposed to a direct effect on the R-loop (Figure 4).

Catalytically dead Cas9 (dCas9) in complex with a guide RNA (gRNA) forms a stable R-loop with a tightly bound protein that is a barrier to the progression of the replication fork, which serves as a model of an RNA polymerase-stalled R-loop barrier [86,87]. The dCas9–gRNA complex arrests replisomes in vitro on both the leading and lagging strand [88]. ScPif1 can work with the CMG-Polε complex to bypass or eject the dCas9–gRNA complex [88]. Surprisingly, neither the replisome nor ScPif1 is efficient at bypassing a dCas9-R-loop block alone, but the combination results in efficient bypass, indicating that ScPif1 does not bypass the block alone [88].

### 3.3. Pif1 Promotes Genomic Stability at Protein Barriers

#### 3.3.1. Pif1 Inhibits Telomerase

Telomere homeostasis is important for mammalian cells, which have a species-specific length. Telomeres shorten with aging, but are preserved in tumors and immortalized cell lines through extension by telomerase to prevent genetic instability and the loss of genetic information [89]. In single-celled eukaryotes such as yeast, telomerase continually maintains telomere length [90]. However, in multicellular eukaryotes, telomere length shortens with each cell division in somatic tissues [91], and the rate of telomere shortening correlates with life-span [92]. This limits tumor formation but also proliferation [93]. A loss of Pif1 is known to result in longer telomeres in a telomerase-dependent manner [8,79], which could be a factor in the increased risk of cancer from a point mutation in hPIF1 [19]. The two important parts of telomerase are the telomerase reverse transcriptase [94] and the RNA component, which acts as a template for the reverse transcriptase to lengthen the telomeres.

ScPif1 is a catalytic inhibitor of telomerase at both telomeres and DSBs [9,10,95], which suppresses gross chromosomal rearrangements [96]. The DNA damage resulting at telomeres from the overexpression of ScPif1 can be rescued by telomerase [97]. The Zakian lab has shown that this occurs because ScPif1 removes telomerase from both telomeric DNA and DSBs [98,99]. However, ScPif1 also promotes the localization of the budding yeast telomerase RNA component, *TLC1*, to the nucleolus, which segregates it from sites of DNA repair [100]. This represses de novo telomere addition, in which telomerase adds a new telomere at a DSB, potentially causing genomic instability and chromosomal aberrations [8]. Preventing the over-extension of telomeres is necessary, but the overexpression of Pif1 often results in a phenotype with telomeres that are too short [9].

Whether Pif1 inhibits telomerase in a telomere length-dependent [99,101] or independent fashion [102] is debatable. ScPif1 binds more efficiently to normal-length telomeres than to short telomeres, resulting in increased telomerase inhibition on normal-length telomeres compared to short telomeres [99]. This suggests that telomere length may be maintained by the preferential lengthening of short telomeres [99]. Telomerase processivity is also enhanced at short telomeres in a Tel1 (ATM) -dependent manner [103]. *pif1-m2* cells exhibit similar phenotypes of telomere elongation to wild-type cells when the telomere length is at least 125 base pairs but increased elongation when the telomere is less than 125 base pairs [102]. However, the frequency of telomere extension was increased at telomeres of all lengths in *pif1-m2* cells, suggesting that ScPif1 inhibits telomerase in a length-independent manner [102]. Telomerase binds preferentially to short telomeres [104,105], suggesting that ScPif1 may reduce telomere length by reducing the frequency of telomere addition at all telomeres and that the preferential extension of short telomeres may be due to the increased association of telomerase with short telomeres.

Interestingly, while the deletion of the NTD on ScPif1 results in a better in vitro inhibition of telomerase in comparison to full-length ScPif1 [29], the overexpression of ScPif1 without the NTD did not cause a telomere length crisis in vivo and was a weaker inhibitor of telomerase in vivo compared to wild-type ScPif1 [29]. The expression of ScPif1 without the NTD also resulted in less telomere shortening than the expression of wild-type ScPif1 [29]. The deletion of the Pif1 signature motif from ScPif1 results in long telomeres, indicating that these residues are important in maintaining proper telomere length and inhibiting telomerase [16].

Like the deletion of ScPif1, the deletion of Rrm3 also results in longer telomeres, but the mechanisms appear to be different. *pif1Δ* yeast have shorter telomeres than *rrm3Δ* yeast, but *pif1Δ rrm3Δ* yeast have a similar telomere length to that of *rrm3Δ* yeast [24]. While ScPif1 inhibits telomerase [9,10,95], Rrm3 associates with telomeric DNA and is involved in fork progression through telomeric and subtelomeric DNA [24]. Rrm3 also does not affect de novo telomere addition in cells with ScPif1, although in cells lacking ScPif1, Rrm3 reduces the de novo telomere addition that occurs in the absence of ScPif1 [24]. ScPif1 inhibits telomeric replication, but Rrm3 promotes semi-conservative replication at telomeres [24], illustrating that these two helicases play different roles at the replication fork and within the cell. Like Rrm3, Pfh1 binds to telomeric DNA [59] and promotes replication through telomeric DNA [106]. Telomere length is decreased in *pfh1Δ* cells (which only survive for a few generations) and increased in cells overexpressing Pfh1 [12,79,106]. Similar to Rrm3, Pfh1 is thought to promote semi-conservative replication through telomeric regions because cells depleted of Pfh1 exhibit fork slowing within telomeric DNA [106,107]. Both Rrm3 and Pfh1 promote replication fork progression through tightly bound protein complexes [107,108]. This could explain why Pfh1 and Rrm3 are needed for replication through telomeric DNA, since telomeric DNA is protected by the protein complexes.

In yeast lacking ScPif1, the de novo telomere addition at DSBs increases dramatically [10,24]. Some evidence suggests ScPif1 is able to distinguish between DSBs and critically short telomeres, which must be processed differently by the cell. DSBs should be repaired without telomerase generating a new telomere at the break, while critically short telomeres must be protected from DSB repair pathways and extended by telomerase. The action of ScPif1 appears to provide at least some discrimination of these structures. It inhibits telomerase at DSBs with short telomeric sequences (less than 34 bp), but not DSBs with long telomeric sequences [109,110]. Mec1 and Rad53, the yeast ATR and CHK2 homologs, respectively, phosphorylate nuclear Pif1 in the presence of a DSB [8]. The phosphorylation of the ScPif1 CTD by Rad53 in response to DNA damage is required for the ScPif1-mediated inhibition of the de novo telomere addition at DSBs but not at telomeres [8].

#### 3.3.2. rDNA Replication Fork Barrier

At the *S. cerevisiae* rDNA locus on chromosome XII, 150–200 repeating units of 35S and 5S rDNA are separated by intergenic spacers containing autonomously replicating sequence (ARS) replication origins between the 5S and 35S loci and a replication fork barrier (RFB) proximal to the 3′ end of the 35S rDNA. The RFB creates a unidirectional 5′-3′ block to the replication fork to prevent head-on collisions between the RNA polymerase I-mediated transcription of the 35S rDNA and the replication fork; thus, 35S rDNA is replicated by replication forks initiated from an ARS in a nearby rDNA repeat in the same direction as transcription [111]. Replication fork stalling at the RFB also serves as an initiator of recombination events between rDNA repeats as a mechanism of maintaining the proper rDNA copy number and identity of rDNA repeats [112,113]. The *S. cerevisiae* RFB is maintained by the binding of Fob1 [112,114]. Fob1 abundance maintains the efficacy of the RFB [115], but Fob1 also serves as a scaffold to recruit Tof1, Csm3 (the *S. cerevisiae* homologs of *H. sapiens* TIMELESS and TIPIN and *S. pombe* Swi1 and Swi3), and DNA topoisomerase I to promote replication fork slowing, transcriptional silencing, and recombination at the RFB [116,117,118,119,120].

Knowledge of the mechanistic role of ScPif1 at the *S. cerevisiae* RFB is limited, but *pif1* mutants lose RFB efficacy and have decreased rDNA recombination events compared to wild-type *S. cerevisiae* [121], suggesting that ScPif1 maintains the RFB through an as-of-yet undescribed mechanism. In contrast, Rrm3 promotes 3′–5′ replication fork progression through the RFB by displacing Fob1 bound at the RFB [108,121,122]. In an Rrm3 N-terminal deletion screen, a lack of residues 134–196 displayed a Fob1-dependent perturbation of rDNA replication, with a broader area of RFB pausing when compared to *rrm3* mutants, suggesting that these N-terminal residues of Rrm3 may be involved in RFB bypass through the replication fork [27]. Notably, it was shown that Rrm3 was not required for replication fork progression through the similar unidirectional replication-blocking *Escherichia coli Ter* replication barrier with bound Tus protein when inserted into the *S. cerevisiae* genome, indicating that Rrm3 does not aid fork progression through all unidirectional protein-bound fork barriers [123]. Rrm3 involvement at the RFB is also associated with the proper replication termination of progressing 3′–5′ forks at the RFB alongside topoisomerase III, Sgs1, and topoisomerase II [124,125]. Further studies of the roles and regulations of Rrm3 and ScPif1 at the *S. cerevisiae* RFB could utilize in vitro replisome progression assays with purified RFB barrier and replisome proteins, as performed previously [126].

A lack of fork progression through the RFB in the absence of Rrm3 has negative consequences on the genomic stability. Stalled replication forks at the RFB increase the ssDNA gaps in *rrm3* mutants [127], and lethality is observed in *S. cerevisiae* with mutations in Rrm3 alongside mutations of other DNA repair and recombination proteins [128,129,130]. Intriguingly, both Rrm3 and ScPif1 have synthetic lethal interactions with the F-box E3 ubiquitin-ligase component Dia2, and given that *dia2* mutants increases rDNA recombination, this suggests an interaction between RFB maintenance, bypass, and protein ubiquitination [128].

Pfh1 also functions at the *S. pombe* RFB. The *S. pombe* rDNA loci are located at both ends of chromosome III and contain 100–150 copies of each rDNA repeat, with an RFB intervening between each repeat [131]. Additionally, the *S. pombe* rDNA loci have four separate repeats, and the repeats are bound by either Sap1 (at the Ter1 site) or Reb1 (at the Ter2 and Ter3 sites) or have unknown protein requirements (at the RBF4 site) [132,133]. The depletion of Pfh1 increases fork stalling at the RFBs [107,134], while the deletion of Swi1, a Sap-1 bound protein required for RFB fork stalling [135,136], prevents replication fork stalling at these barriers (Steinacher et al., 2012). This suggests that Pfh1, similar to Rrm3 and unlike ScPif1, may disrupt Sap1 and Reb1 or other protein binding at the *S. pombe* RFBs to promote replication fork progression through the rDNA loci [107,134].

#### 3.3.3. Highly Transcribed Genes

Additionally, conflicts between the replication and transcription machineries may cause replication fork stalling [47]. tRNA genes (tDNAs) are highly transcribed, and the stalling of DNA replication occurs in *rrm3 S. cerevisiae*, irrespective of the relative orientations of the complexes (co-directional and head-on collisions) [47,122,137,138]. In *rrm3* mutants, ScPif1 promotes the progression of the replisome through tRNA genes [47,137,138]. Highly transcribed RNA polymerase II genes also cause the pausing of the replication fork, but this is not enhanced in *rrm3 S. cerevisiae* [47]. Although head-on and co-directional collisions can both cause replication fork pausing, head-on collisions induce replication fork arrest more frequently, and Rrm3 and ScPif1 appear to affect fork pausing in both orientations equally [137]. However, the specific roles of Rrm3 and ScPif1 during collisions with the transcriptional machinery are under debate. Data from one group indicate that Rrm3, and, to a lesser degree, ScPif1 decrease the pausing of the replication fork at tRNA genes by resolving R-loops [138]. However, another group found that replication forks lacking Rrm3 arrest at tRNA genes in an R-loop-independent manner [137]. The reasons for these discrepancies are unclear, so the role of R-loops in pausing at tRNA genes is still an open question. *S. pombe* lacking Pfh1 also show increased pausing at tDNAs [107]. A mutation in the promoter of tDNAs that prevents transcription abolishes fork pausing in both WT and *rrm3Δ* cells [122]. While replication fork pausing during transcription occurs naturally [139], it is more pronounced in cells depleted of Pfh1 [107] and at certain sites in *rrm3Δ* cells [122].

## 4. Lagging-Strand Synthesis

During the synthesis of the lagging strand, DNA polymerase α-primase synthesizes an RNA primer to form an RNA/DNA hybrid [140]. The lagging-strand polymerase, DNA polymerase δ, in complex with PCNA extends the primers to produce short daughter strands called Okazaki fragments from the RNA primers, which vary from ~100 nt in humans to ~250 nt in *S. cerevisiae* [141]. Okazaki fragments have short 5′-flaps of ssDNA/RNA that overhang and are cleaved by FEN1 then ligated by DNA ligase I. Pif1 has been shown to interact with predominantly DNA polymerase δ, which extends Okazaki fragments initiated by polymerase α-primase [142].

**Figure 5:**
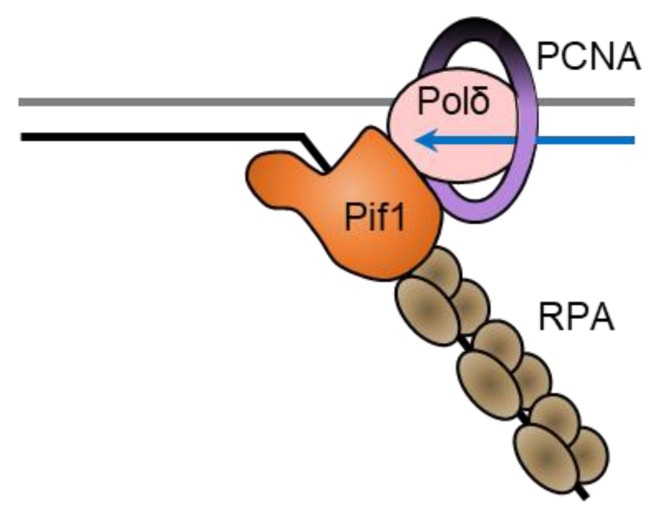
Extension of the 5′-flap of an Okazaki fragment by Pif1 provides a binding site for RPA that makes it resistant to cleavage by FEN1.

Rarely, strand displacement synthesis by the ScPif1-PCNA-Polδ complex extends the flap before cleavage (Figure 5) [143]. These long flaps can be cleaved by FEN1 before the binding of RPA, but if they are lengthened sufficiently to allow RPA binding, they are resistant to cleavage by FEN1 [143,144,145] but can be cleaved by Dna2 [146,147,148]. In this two-nuclease pathway of Okazaki fragment processing, Dna2 cleaves the long flap generated by ScPif1-Polδ, producing a short flap which RPA cannot bind to [143] and FEN1 can then cleave [144,145,149].

The deletion of Dna2 is lethal [150] and activates the DNA damage response [151]. *pif1Δ* rescues the lethality of *dna2Δ*, suggesting that the requirement for Dna2 results from the action of Pif1 [152]. Similarly, *pfh1* mutations suppress the temperature-sensitive phenotype of a *dna2* mutant in *S. pombe* [153]. Dna2-depleted *S. cerevisiae* accumulate ssDNA flaps that likely result from strand displacement synthesis by Polδ and ScPif1 [151]. This suggests that the accumulation of long flaps generated by ScPif1-Polδ during Okazaki fragment processing is toxic, and the processing of these long flaps is an essential activity of Dna2.

Both ScPif1 and Rrm3 enhance the processivity of Polδ, such that it synthesizes to the midpoint of the nucleosome as opposed to the proximal edge of the nucleosome, suggesting that these helicases remove or reposition the nucleosome or partially unwrap the DNA from the nucleosome to allow synthesis to continue [137]. The transcription factors Rap1, Abf1, and Reb1 interact with the DNA at positions that coincide with Okazaki fragment ends, suggesting that their binding induces Polδ dissociation and Okazaki fragment termination [154]. Rap1 binds to telomere replication forks and slow them in a sequence- and concentration-dependent manner, as well as inhibiting lagging-strand replication behind the fork in vitro [155]. Rap1 inhibits strand displacement synthesis by Pol δ and ScPif1, but not RPA or Dna2, and stimulates Pol δ to extend the 5′ flap and bypass Rap1 [156]. Douglas and Diffley revealed that Pif1 promotes the bypass of Rap1 in a Pol δ-independent manner, and the authors suggest that Pif1 displaces Rap1 in front of the replication fork [155].

Reb1 is also a block to Pol δ strand displacement synthesis, even with RPA stimulating Pol δ synthesis [157]. While the forward orientation of Reb1 is a greater block to replication than its reverse orientation, Pif1 can remove Reb1 downstream of Pol δ [157]. Similar to the protein blocks in Okazaki fragment processing, nucleosomes produce a barrier that Pif1 and not RPA can resolve for Pol δ synthesis [157]. In vitro assays conducted by the Galletto lab suggest that Pif1 removes the nucleosomal barrier rather than the barrier being pushed off the end of the dsDNA substrate [157].

## 5. Break-Induced Replication

**Figure 6:**
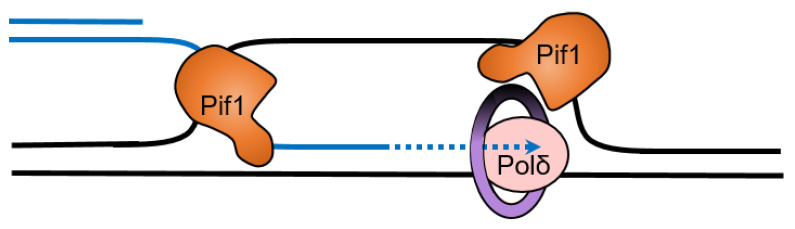
Pif1 promotes break induces replication with Polδ and PCNA. A second Pif1 may unwind the nascent strand to resolve the D-loop and reduce topological stress.

Break-induced replication (BIR) is a type of homologous recombination involved in the repair of one-ended double-strand DNA breaks at collapsed replication forks. BIR is also responsible for the alternative lengthening of telomeres (ALT) [158,159,160], mitotic DNA synthesis (MiDAS) [161], and the maintenance of the mitochondrial DNA networks in the human heart [162]. BIR begins with processing the DNA end into 3′-ssDNA tails onto which Rad51 recombinase is loaded [163]. Then, Rad51 initiates the strand invasion of the 3′-tail to form a displacement loop (D-loop). Unlike canonical S-phase DNA synthesis, BIR involves conservative DNA synthesis by Polδ in a migrating D-loop [164]. Yeast, drosophila, and human cells depleted of Pif1 are deficient in BIR, but the depletion of Rrm3 does not affect BIR [76,142,165]. It is possible that two Pif1 molecules participate in BIR, with one at the leading edge of the migrating D-loop, which stimulates strand displacement synthesis by Polδ through its interaction with PCNA [39]. A second Pif1 at the back of the migrating D-loop may release the nascent strand to alleviate topological constraint [142] (Figure 6).

Hundreds of kilobases of DNA can be synthesized in BIR [166], but this promotes genomic instability [167]. The synthesis of the leading and lagging strands is asynchronous in BIR, which leads to the accumulation of mutation-prone ssDNA [164]. The mechanism of BIR also leads to a loss of heterozygosity. Copy number variations, translocations, and the expansion of repetitive elements can also result from BIR due to out-of-register strand invasion after replication fork stalling [168,169,170]. Thus, BIR allows cells to survive replication-induced DSBs, but it results in an accumulation of genomic anomalies that are hallmarks of cancer genomes. Overall, BIR appears to protect the genome because the localization of hPIF1 to common fragile sites that are prone to replication stalling induces BIR and reduces genomic instability [165]. In addition, a cancer-associated hPIF1 mutation (L319P) defective in BIR is associated with increased DNA damage [165]. Thus, the effects of BIR on genome stability are not straightforward.

## 6. Fork Convergence during Replication Termination

ScPif1 and Rrm3 aid in the fork convergence of budding yeast replication machinery in vitro; thus, the depletion of either ScPif1 or Rrm3 results in delayed fork convergence independent of type II topoisomerase activity [171]. The two Pif1 helicases likely unwind the lagging strand template, aiding the CMG (Cdc45-MCM-GINS) helicase, which moves in the 3′-to-5′ direction and unwinds the leading strand template [171]. How ScPif1 and Rrm3 promote fork convergence is not clear. It is possible that ScPif1 and Rrm3 enhance the ability of the CMG helicases of converging replisomes to pass each other to allow synthesis to complete, although this seems unlikely because the converging CMG would be bound to opposite strands, and CMG can bypass blocks on the other strand [172,173]. ScPif1 and Rrm3 may also promote fork convergence by removing inactive Mcm2-7 double hexamers from the region of fork convergence; however, ScPif1 and Rrm3 reduce the formation of late replication intermediates even when only one Mcm2-7 double hexamer is loaded on the template [171]. It is also possible that ScPif1 and Rrm3 relieve the torsional strain generated by converging replisomes and prevent the stalling of replication termination by late termination intermediates [171]. Why this would be a function of ScPif1 and Rrm3 instead of topoisomerases is unclear.

## 7. Mitochondrial DNA Replication

Pif1 localizes to the mitochondria and the nucleus in both human and yeast cells [4,174] and was initially identified due to defects in the recombination of ρ^−^ (petite mutant) *Saccharomyces cerevisiae* [3]. The importance of Pif1 for mitochondrial DNA (mtDNA) repair [3,175] and for the mitochondrial DNA (mtDNA) maintenance at elevated temperatures [4] in *S. cerevisiae* have been known for quite some time. More recently, mice lacking PIF1 have been shown to develop mitochondrial myopathy due to respiratory chain deficiency and mtDNA deletions, indicating that mPIF1 is also critical for mtDNA maintenance [176].

**Figure 7:**
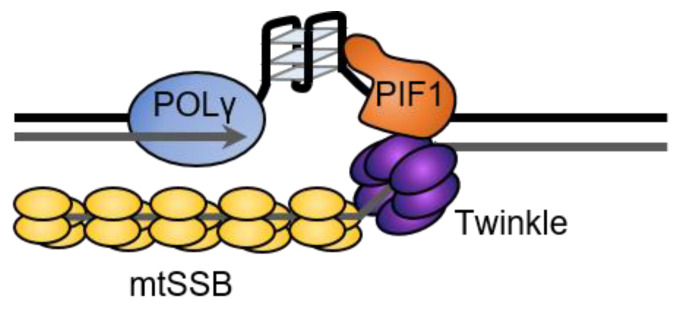
Pif1 promotes synthesis through G4 sequences by yeast and human mitochondrial polymerases. The role of Pif1 may be to unfold G4 structures that fold after the unwinding of the duplex before synthesis occurs.

In addition to its roles in mtDNA repair, ScPif1 has been suggested to be part of the mitochondrial replisome, where it could aid in replication of G4s in mitochondrial DNA [15]. Synthesis by the yeast and human mitochondrial replicative polymerases, Mip1 and POLγ, is blocked by G4s in vitro [66,67]. However, Pif1 stimulates synthesis by Mip1; POLγ; and another mitochondrial polymerase, PRIMPOL, at G4 sequences, suggesting that Pif1 promotes the progression of the mitochondrial replication fork through G4 structures [66,67]. The mechanism of this stimulation is unknown but, based on the 5′-to-3′ directionality of Pif1 and the movement of the polymerase 3′-to-5′ along the template, Pif1 is likely to unfold the G4 structure from the opposite direction of the replisome progression (Figure 7). This is consistent with the design of in vitro synthesis experiments with Pif1 and POLγ [66], Pif1 and Mip1 [67], and Pif1 and Polδ [40,67] and is similar to the mechanism proposed by FANCJ for the unfolding of G4s that form between MCM and the polymerase during nuclear replication [177].

## 8. Regulation of Pif1 Activity

Regulation of Pif1 expression and activity is important, since the DNA helicase is involved in many aspects of replication [178] and has been linked to breast cancer [19] and obesity [179]. Each of the three domains (NTD, helicase, CTD) of ScPif1 contains at least one lysine acetylation site regulated by the acetyltransferase NuA4 and the deacetylase Rpd3 [2], which may cause a conformational change in the protein [2]. Interestingly, the overexpression of ScPif1 is toxic [4,97] and the acetylation of the NTD intensifies its toxicity [2]. In contrast, the deletion of the NTD alleviates the toxicity [29]. The mutation of NuA4 decreases the toxicity of Pif1 overexpression, and *S. cerevisiae* without Rpd3 experienced higher levels of toxicity with Pif1 overexpression [2]. This is likely due to the improved helicase-catalyzed unwinding of acetylated ScPif1 for both forked and tailed substrates [2]. This improvement of unwinding occurs due to an increase in the processivity and not an increase in the rate.

In *S. cerevisiae* strains with impaired replication, telomere lengthening occurs in a telomerase- and BIR-dependent manner [180]. DNA damage signaling results in the phosphorylation of ScPif1 by Mec1 and Rad53, the yeast ATR and Chk2 homologs, respectively, to phosphorylate nuclear Pif1 at the sequence TLSSAES (T763-S769) [180]. Interestingly, the phosphorylation of the same site in response to DNA damage is required for ScPif1 to inhibit de novo telomere addition at DSBs [8]. The replacement of the serines and threonines in the phosphorylated motif with the unphosphorylatable alanines or phosphomimetic aspartic acid had no effect on telomere length, but the alanine mutant increased de novo telomere addition, similar to that seen in *pif1-m2 cells* [8].

In *pif1-m2 yeast*, which have no nuclear Pif1, and *pif1-C-18A*, where all the serines and threonines from T763 to the end are mutated to prevent phosphorylation, a greater frequency of de novo telomere addition was found, suggesting that the phosphorylation of the ScPif1 C-terminus plays a role in inhibiting telomerase at DSBs [8]. This study created two other mutant strains, replacing all of the threonines and serines from T763-S769 with either non-phosphorylatable residues (*pif1-4A*) or the phosphomimetic residues *pif1-4D* [8]. Both were able to inhibit telomerase at telomeres, but the *pif1-4A* strain could not inhibit de novo telomere addition at DSBs and was shown by chromatin immunoprecipitation (ChIP) to localize to DSBs, indicating that this motif is necessary for the proper Pif1 inhibition of telomerase at DSBs [8]. Additionally, Mec1 and Rad53 phosphorylate nuclear Pif1 in the presence of a DSB [8], but the DNA damage produced by the overexpression of ScPif1 in telomeres can be rescued by telomerase [97]. This illustrates the complex checks-and-balance system used for telomere maintenance.

## 9. Conclusions

The Pif1 family helicases are conserved from bacteria to yeast and humans. The helicase plays a major role in maintaining cellular survival and fitness due to its roles in resolving replication barriers, facilitating proper lagging-strand synthesis, and promoting break-induced replication. The unique role of Pif1 in cellular health highlights the overwhelming number of regulatory pathways and mechanisms the cell uses for proper DNA replication and transcription. While the mutation of the signature motif of Pif1 results in a phenotype leading to a higher risk of cancer, many studies indicate that the depletion of Pif1 also results in higher genomic instability and mutations that could lead to cancer.
